# Prevalence and Correlates of Mental Health Issues Among University Students in Punjab, Pakistan: Insights into Academic Performance and Psychological Well-Being

**DOI:** 10.3390/healthcare14101421

**Published:** 2026-05-21

**Authors:** Nauman Ali Chaudhry, Rubeena Zakar, Gulzar H. Shah, Alexander Kraemer, Bushra Shah

**Affiliations:** 1 Department of Public Health, University of the Punjab, Lahore 54590, Pakistan; naumanali.iscs@pu.edu.pk (N.A.C.); rubeenaz.iscs@pu.edu.pk (R.Z.); 2 Jiann-Ping Hsu College of Public Health, Georgia Southern University, Statesboro, GA 30460, USA; bs06779@georgiasouthern.edu; 3 School of Public Health, Bielefeld University, 33501 Bielefeld, Germany; alexander.kraemer@uni-bielefeld.de

**Keywords:** mental health, university students, academic performance, depression, psychological well-being

## Abstract

**Background/Objectives:** Mental health problems are common among university students and are more consistently associated with dissatisfaction with academic performance than with low grades alone. This study examined the prevalence and determinants of perceived stress, depressive symptoms, and low psychological well-being among university students in Punjab, Pakistan, and assessed their association with academic performance. **Methods:** A cross-sectional survey was conducted among students aged 15 to 29 years at three public universities in Punjab, Pakistan. A total of 1308 questionnaires were completed, yielding a response rate of 91.4%. This study uses data collected in 2015 as a pre-COVID historical baseline, providing valuable insights into student mental health before the global pandemic. This temporal context offers a benchmark for future comparative studies, especially when assessing the mental health impact of COVID-19 on university students. Data were analyzed using SPSS with descriptive statistics, chi-square tests, binary logistic regression, and multinomial logistic regression. **Results:** The findings revealed that perceived stress and depressive symptoms were prevalent, with 54.9% of students reporting high levels of stress (mean PSS score = 27.6, SD = 8.3), and 44.2% experiencing depressive symptoms (mean M-BDI score = 33.8, SD = 16.2). Female students exhibited higher stress and depressive symptoms compared to male students. Year of study was also a factor, with second- and third-year students experiencing more stress than their final-year counterparts (*p* < 0.05). Financial strain was associated with poorer mental health outcomes; 62% of students who reported inadequate financial support also reported higher stress levels (*p* < 0.05). In contrast, students with sufficient financial resources had lower odds of experiencing stress and depressive symptoms (AOR = 0.55, *p* < 0.05). Additionally, students living in university or private hostels reported better psychological well-being than those living at home (AOR = 0.47, *p* < 0.01). Mental health issues, particularly high stress and depression, were more strongly linked with academic dissatisfaction than low grades alone, with students in the “low grades and unsatisfied” group exhibiting higher odds of mental health problems (AOR = 2.30, *p* < 0.05). **Conclusions:** Mental health problems were common among university students and were associated with poorer academic experiences, particularly dissatisfaction with academic performance. Universities should strengthen accessible mental health support through counseling services, stress-management programs, and stigma-reduction initiatives.

## 1. Introduction

Previous studies have highlighted the increasing prevalence of mental health disorders among university students in various countries, with evidence suggesting that approximately 25% of students experience mental health challenges. According to the World Health Organization (WHO), mental health disorders account for a substantial proportion of the global burden of disease, with university students being particularly vulnerable [1]. The WHO reports that approximately 15% of young adults suffer from mental health disorders, a statistic that is reflected in various studies across different regions [1,2]. The growing use of measures such as years lived with disability and disability-adjusted life years has highlighted the substantial population burden of mental health conditions relative to other major health problems [3,4]. Despite this, the burden of mental health conditions worldwide is onerous to determine because they primarily affect physical health indirectly and usually aggravate other health problems; hence, their effect on the overall well-being is not easy to measure.

Studies indicate that university students are more vulnerable to mental health problems than other age groups [5,6]. Students in this population face several stressors that may contribute to the development or worsening of mental health conditions. In Pakistan, cooperation between the education and health sectors remains limited, and sustainable institutional frameworks to support student mental health are still lacking [7]. The absence of comprehensive campus mental health programs further exacerbates these challenges, particularly for students with undiagnosed or poorly recognized mental health problems. Adjusting to university life is a complex process in itself. Students are exposed to multiple stressors, including cultural adjustment, academic pressures, career expectations, social demands, financial difficulties, and competing personal responsibilities [5,8,9]. These stressors can place students at increased risk of psychological distress or depression, which, if left unaddressed, may lead to more serious mental health problems [10].

Academic pressure basically goes hand in hand with mental health issues within the university student population [11]. As students face immense pressures, studying academic stress is crucial because it is associated with their adaptation to university life [12]. Although low amounts of stress, also known as eustress, can lead to positive academic results, too much distress can lead to adverse academic outcomes, decreased self-esteem, and the development of adverse mental health effects in the long-term [13,14]. Research has established that many university students (40 percent) access health systems because of anxiety, poor concentration, and stress. These neurotic students demonstrate a considerable level of neuroticism. Still, they do not necessarily perform academically worse than others, and this fact indicates that the connection between mental health and academic performance is complex [15]. Surtees et al. (2002) also discovered that when other factors were correct, depression and anxiety did not necessarily influence the chances of the students being awarded a first-class degree [16]. In other studies, it has been established that students with high scores on the General Health Questionnaire (GHQ) due to distress were not at increased risk of failing their first-year exams [17]. In historical studies, a weaker association between stress and academic performance has been reported, perhaps because academic environments are less competitive, annual examinations are used rather than a semester system, and fewer academic demands are imposed [18].

Academic performance influences mental health in multiple ways, including stress, self-esteem, social pressures, and support systems, with both positive and negative outcomes. There is a tendency to link high academic success with better career opportunities, higher wages, and greater social mobility. However, academic achievement pressure may have severe adverse effects, elevating stress levels and exacerbating mental health issues among students. Other research has shown that students with high perceived stress and depressive symptoms tend to achieve lower results, particularly when they are dissatisfied with their grades, regardless of their actual performance [18,19].

Mental health is not the only factor that determines academic performance. The academic setup, family, developmental background, and academic tasks also contribute significantly to academic success. Learners from lower socioeconomic classes also face additional challenges that may adversely affect their academic achievement. The culture of competitiveness in academic settings focuses more on grades, which makes seeking counseling to deal with a mental health problem carry a lot of stigma. The fear of students that their mental health issues will be perceived as their weakness and their inability to stick to their studies may lead them to avoid getting their mental issues examined and addressed [19,20,21,22].

Mental health issues can have profound impacts on students’ lives, affecting their personal lives and their future success. Living with mental health issues is not only an individual issue but also a societal one. Health-seeking behavior is low in Pakistan, even though mental health disorders are not only difficult to understand but also generally unknown in their severity. It is acknowledged that, despite access to medical information, there is a set of stereotypes about mental health patients, which leads to a star constellation of unmet mental health needs and risk factors [20]. In addition, the economic situation of many university students, the stress associated with studies, and their psychological development make the problem even more difficult. The issues also affect the developing nations, especially Pakistan, which experiences massive economic insecurity and a low rate of economic growth, exacerbating the mental health issues of students. The level of health literacy in Pakistan (26.2%) is also low, with traditional non-scientific followings of treating mental health issues usually employed, thereby restricting the students from seeking adequate treatment [21].

This study offers a novel distinction between objective academic performance (grades) and subjective academic satisfaction, an underexplored area in the literature on student mental health. By examining both, this research provides a deeper understanding of how students perceive their academic success and how it influences their mental health. These insights have broad implications for designing future interventions and policies to improve student well-being.

This study adds to the literature by focusing on an underrepresented context: students enrolled in public universities in Punjab, Pakistan. By exploring both objective academic performance and subjective academic satisfaction, we offer a more comprehensive understanding of how mental health issues intersect with academic life. Drawing on the Stress-Coping Model, this study provides valuable insights into how students’ coping strategies and perceptions of academic stress influence their psychological well-being. Our findings contribute to the broader understanding of student mental health in a South Asian context, which is often underexplored in existing research.

The data collected for this study represent a pre-COVID university context (2015) and serve as a valuable historical baseline. While the global landscape of student mental health has undergone significant shifts due to the COVID-19 pandemic, these pre-pandemic findings remain highly relevant. They offer essential insights into the mental health and academic experiences of university students before the pandemic, providing a benchmark for future comparative studies on the long-term impact of the pandemic on student well-being.

Recent studies have shown that the COVID-19 pandemic has had a profound impact on student mental health, with increased levels of anxiety, depression, and stress reported globally. These studies highlight the importance of examining mental health in the current context to understand the evolving challenges faced by university students. This study contributes to the ongoing discourse by providing a historical baseline for understanding students’ mental health in a pre-pandemic environment, which can later be compared with current data in future studies.

This study examined the prevalence and determinants of perceived stress, depressive symptoms, and low psychological well-being among university students in Punjab, Pakistan, and assessed their association with academic performance. A particular contribution of this study is its distinction between low grades and dissatisfaction with academic performance, allowing a more nuanced assessment of how mental health relates to students’ academic experience.

## 2. Theoretical Framework

### Stress-Coping Model

Perceived stress and depression, as well as low well-being rates among university students, are serious mental issues that distort not only their psychological state but also their academic output. To elucidate these relations, this paper draws on the Stress-Coping Model [23,24], which examines how individuals perceive and cope with stress.

According to this model, stress arises when students experience a gap between the demands placed on them (e.g., exam pressure, financial needs, social demands, etc.) and their capacity to respond [23,24,25,26,27]. According to Folkman and Lazarus, coping is divided into problem-focused and emotion-focused coping: dealing directly with the stressor (e.g., seeking academic assistance, managing time) is problem-focused, and handling the emotional response (e.g., avoidance, denial) is emotion-focused [24]. It is the type of coping strategy and the cognitive appraisal of the stressor that influence the impact of stress on mental health and academic achievement [23].

In the present study, the Stress-Coping Model was operationalized by mapping measured variables onto demands, resources, appraisals, and mental-health outcomes. Academic pressure, low grades, grade dissatisfaction, and insufficient financial support were treated as stressors or indicators of perceived academic and socioeconomic demands. Sufficient financial support and living arrangements were treated as contextual resources that may buffer stress. Academic satisfaction was treated as a cognitive appraisal of students’ academic experience, whereas perceived stress, depressive symptoms, and psychological well-being were treated as mental-health outcomes. This mapping guided the selection of covariates in the regression models and the interpretation of associations among academic, socioeconomic, and mental-health variables.

While previous studies have explored mental health issues in university students, most research has concentrated on limited aspects, often overlooking the socio-economic factors that may play a significant role. There is a notable gap in the literature when it comes to understanding how financial stress, housing conditions, and other socio-economic variables affect students’ mental health, especially in developing countries like Pakistan. This study aims to address this gap by focusing on these important contextual factors and their influence on students’ mental well-being and academic performance in Punjab, Pakistan.

This research aims to investigate the prevalence of mental health issues among university students in Punjab, Pakistan, with particular attention to perceived stress, depressive symptoms, and psychological well-being. It also explores the relationship between mental health and academic performance, focusing on socio-economic factors such as financial support and living arrangements.

To ensure consistency between the theoretical framework and the empirical analysis, the study variables were organized according to the Stress-Coping Model and then used to guide covariate selection and interpretation of the regression models.

## 3. Materials and Methods

### 3.1. Enrollment of Respondents

Data were collected through a cross-sectional survey administered to students at three public universities in the province of Punjab, Pakistan. According to the Government of Pakistan, the youth population is aged 15–29; hence, respondents were sampled from this age group [22]. Doctoral students were excluded from the sample because their age and socio-economic status differ, and they cannot be considered representative of the target population [22]. To ensure the sample was well exposed to city and university life, the inclusion criteria required respondents to have taken at least 2 semesters or 1 year at the university. This criterion was meant to ensure that the sample comprises students with proper knowledge of the university environment.

This study employs a quantitative research design, grounded in the Stress-Coping Model. The model guided the selection of key variables, including problem-focused and emotion-focused coping, as well as the inclusion of academic satisfaction and mental health outcomes. These variables were assessed through a survey designed to capture students’ academic experiences and their coping strategies. The model’s emphasis on the appraisal of stress and the coping mechanisms employed by individuals provided a theoretical framework that underpinned the research design. This approach enabled a comprehensive analysis of how students’ perceptions of academic demands and their coping responses influence their mental health.

### 3.2. Sampling Procedures

The sampling method used was multistage cluster random sampling, in which respondents were selected. The following three public universities were randomly selected in the first step to ensure representation of both the province’s geographic and cultural centers. A proportional sampling frame for each university was then prepared based on the number of students across faculties and departments. Classes in each department were then discussed as a cluster, and all students in that cluster were included in the sample.

To achieve optimal representation, data were collected across departments and degree programs, among undergraduate and postgraduate students, in both regular and self-supporting sessions. Although the number of representatives by gender at each university was nearly equal, the sample was designed to be representative of both genders. The fieldwork was conducted from January to 28 March 2015.

### 3.3. Sample Size

The sample size was determined using the formula *n* = *N*/(1 + *N*(e)^2^). The level of precision (e) was assumed to be ±3% because the actual population value was estimated to have varied during the period when the data were primarily collected. The sample size, computed using the formula, was 1087 (See Table 1). To account for nonresponse, 20% was added to the estimated sample size of 1087, yielding a target sample of 1308.

### 3.4. Study Design

This study used a cross-sectional quantitative research design.

### 3.5. Tools for Data Collection

This study used a self-completed, pre-coded questionnaire, modified from the Cross-National Student Health Study (CNSHS) in Germany. Owing to the contextual and cultural nature of the questions used in the CNSHS questionnaire, which was developed among European university students [29], a number of these questions were also culturally and contextually specific to the students. Given disparities between European and Pakistani cases, the draft questionnaire was revised based on responses from public health experts, university administrators, and psychologists in Pakistan. Also, the CNSHS questionnaire was accompanied by various tools that enabled its adaptation to align with the research questions of the present study. The questionnaire was written in English, since it is the language of instruction at the chosen universities. The initial section of the revised questionnaire was used to collect respondents’ demographic data.

### 3.6. Measures

#### 3.6.1. Key Determinants

The main correlates examined in this study included age, sex, marital status, place of birth, monthly family income, employment status, university, academic faculty, degree/program, type of degree, year of study, living arrangement, and sufficiency of financial support. Their reported effect informed the selection of these variables to examine mental health in a university setting, particularly in South Asia.

#### 3.6.2. Psychological Well-Being Index (WHO-5)

The World Health Organization’s (WHO-5) Well-Being Index is a well-established tool designed to assess subjective well-being, focusing on aspects such as positive mood, vitality, and general interest in activities over a two-week period [30]. It consists of five questions, in which respondents rate their experiences on a 6-point scale ranging from 0 (never) to 5 (all the time). The total score can range from 0 to 25, with lower scores (below 13) indicating lower psychological well-being.

#### 3.6.3. Perceived Stress Scale (PSS)

The Perceived Stress Scale (PSS) by Sheldon Cohen measures how unpredictable, uncontrollable, and overloaded an individual perceives their life to be [31]. This 14-item tool provides a short version to assess perceived stress, with respondents rating their experiences on a five-point scale ranging from 0 (never) to 5 (very often). Scores on the PSS range from 0 to 56, with higher scores indicating greater perceived stress. Those scoring above 28 are classified as stressed [31].

#### 3.6.4. Modified Beck Depression Inventory (M-BDI)

The Modified Beck Depression Inventory (M-BDI) is a 20-item scale designed to measure depressive symptoms in individuals [32]. Each item is rated on a five-point scale from 0 (never) to 5 (almost always), with the cumulative score ranging from 0 to 100. A score above 35 indicates clinically relevant depression [32].

#### 3.6.5. Construct Clarification

Perceived stress refers to students’ appraisal of life demands as unpredictable, uncontrollable, or overwhelming. Depressive symptoms refer to mood-related symptoms measured by the M-BDI. Academic pressure refers specifically to perceived demands arising from academic workload, expectations, and performance concerns. Academic satisfaction reflects students’ subjective appraisal of their academic outcomes and is distinct from objective GPA.

#### 3.6.6. Subjective Performance

Subjective performance refers to how students perceive their academic achievements relative to their expectations and standards. It is assessed using a Likert scale, where students rate their perceived academic performance from very poor to excellent.

#### 3.6.7. Academic Pressure

Academic pressure refers to the stress and demands students feel due to academic expectations, workload, and societal pressures. It was assessed using a Likert scale, and previous studies have reported strong reliability for this measure, with test–retest reliability of 0.90 over two weeks [11,12]. This suggests that the scale is a stable and valid measure of academic pressure among university students.

#### 3.6.8. Satisfaction with Grades

Satisfaction with grades was operationalized as a student’s degree of satisfaction with their academic performance. It was measured as a dichotomous variable indicating whether students were satisfied or dissatisfied with their grades. The construct is meant to measure cognitive and emotional responses to academic performance and is not restricted to GPA alone, but also to personal satisfaction with academic achievements.

#### 3.6.9. Urdu Versions and Indicators

Both the WHO-5 and PSSs were translated into Urdu to ensure accessibility for Pakistani populations. The indicators in these scales assess various emotional and psychological states, such as happiness, calmness, energy levels, and feelings of being overwhelmed or in control. Similarly, the M-BDI includes indicators related to mood, interest in activities, and overall emotional well-being.

The questionnaire was adapted from the Cross-National Student Health Study and reviewed for contextual relevance by public health experts, university administrators, and psychologists in Pakistan. The study measures were reviewed to ensure conceptual equivalence, cultural appropriateness, and relevance to public university students in Punjab. Internal consistency in the present sample was acceptable to good for the three main mental-health measures: WHO-5 Well-being Index, Cronbach’s α = 0.78; Perceived Stress Scale, Cronbach’s α = 0.74; and Modified Beck Depression Inventory, Cronbach’s α = 0.81. These values indicate that the items within each scale showed adequate internal consistency in the current sample. However, because formal psychometric validation of all adapted measures among public university students in Punjab was limited, this issue is acknowledged as a limitation of the study.

In the data analysis, the constructs of well-being, perceived stress, and depression were categorized into low and high categories based on specific scoring thresholds derived from the standardized scales. These measures collectively offer a comprehensive assessment of psychological well-being, stress, and depressive symptoms, providing a basis for targeted mental health interventions for university students.

#### 3.6.10. Scoring and Categorization of Mental-Health Measures

The WHO-5, PSS, and M-BDI scores were analyzed both descriptively as continuous scores and categorically using established thresholds. Low psychological well-being was defined as WHO-5 < 13, high perceived stress as PSS > 28, and depressive symptoms as M-BDI > 35. These cut-offs were used to estimate the prevalence of students at risk and to facilitate binary logistic regression models. However, dichotomizing continuous scales may reduce information, statistical power, and variation within categories. For this reason, mean scores and standard deviations are also reported. Analyses using continuous versions of the mental-health scales yielded substantively similar patterns of association, supporting the robustness of the findings to alternative specifications.

### 3.7. Ethical Considerations

Mental health issues remain socially stigmatized in many societies, including Pakistan [33]. Since this study focused on students’ mental health, the primary ethical consideration was ensuring respondents’ anonymity. To achieve this, no personally identifiable information—such as names, registration numbers, or contact details—was collected during data collection. Informed consent was obtained from each respondent both verbally and in writing. Written consent was documented through a statement at the beginning of the questionnaire, indicating that participation was voluntary and that respondents could withdraw at any time [34].

This study was part of the doctoral dissertation of the first author. It was approved by the ethical review board at the Faculty of Behavioral and Social Sciences, University of the Punjab (No: D/668/FBSS).

### 3.8. Missing Data Handling

Missing data for the Perceived Stress Scale (PSS) accounted for approximately 16% of the sample. These missing values were handled using multiple imputation, a statistical method that generates multiple plausible datasets by replacing missing values with estimates based on observed data patterns. This approach ensures the accuracy of the results while maintaining the integrity of the sample.

Missing data for the Perceived Stress Scale (PSS) accounted for approximately 16% of the sample. These missing values were handled using multiple imputation by chained equations (MICE) in SPSS. This method generates multiple plausible datasets by estimating missing values based on observed data patterns, ensuring that all available data is used efficiently. The imputation model included all variables used in the analytic models, such as sociodemographic characteristics, academic variables, and mental health scale scores. A total of 20 imputed datasets were generated, and pooled estimates were reported. Complete-case analyses were conducted as a sensitivity check, and results showed similar patterns. The final analytic sample size after imputation was 1277.

### 3.9. GPA Cut-Off Justification

The GPA categorization into specific brackets (≤3.00, 3.01–3.30, 3.31–3.70, 3.71–4.00) was based on common institutional grading practices. These cut-off points correspond to standard GPA thresholds used by many universities in Pakistan to differentiate academic performance levels. The categories were chosen to reflect both moderate and high levels of academic achievement, ensuring that the analysis captured a broad range of students’ academic experiences.

### 3.10. Data Analysis

Data were analyzed using the Statistical Package for the Social Sciences (SPSS version 22), a widely used statistical software package. The SPSS software is produced by IBM, based in the United States. The descriptive statistics for the quantitative variables were presented as means and standard deviations, while those for the categorical variables were presented as frequencies and percentages. The chi-square test was used to assess the relationship between respondent characteristics and mental health issues regarding academic performance. Binary logistic regression was used for perceived stress, depressive symptoms, and low psychological well-being because these outcomes were categorized as binary variables. Multinomial logistic regression was used for academic performance because the dependent variable consisted of four nominal categories combining grade level and satisfaction with grades: low grades/unsatisfied, low grades/satisfied, high grades/unsatisfied, and high grades/satisfied. The high grades/satisfied group was used as the reference category. Models were adjusted for sex, place of birth, university, academic faculty, degree/program, and living arrangement. Multinomial logistic regression was then used to assess the association between mental health indicators and four categories of academic performance, using high grades with satisfaction as the reference category. Adjusted models controlled for sex, place of birth, university, academic faculty, degree, and current place of living. The dependent variable, academic performance, was categorical with multiple levels, allowing exploration of how various independent variables (including mental health factors) influenced the likelihood of students being assigned to different academic performance categories.

## 4. Results

### 4.1. Prevalence of Mental Health Issues and Socio-Demographic Characteristics

The respondents were aged 16–28 years (mean = 21.5; standard deviation (SD) = ±1.7). The sample was almost equally divided between males and females, with 657 males (50.2%) and 651 females (49.8%) (see Table 2).

Most of the respondents in the sample (78.4%) were in their second year of study, followed by 14.9% in their third year, and only 6.7% in their fourth year. Regarding current living arrangements, 64.8% of respondents reported living in their homes, whereas 34.2% reported living in university hostels (22.5%) or private hostels (11.7%).

### 4.2. Prevalence of Mental Health Issues

#### 4.2.1. Psychological Well-Being

Most of the respondents (81.0%) reported high psychological well-being, whereas only 19.0% of the respondents had low psychological well-being. The data showed that the mean score of all respondents was 16.5 (SD = 4.9).

#### 4.2.2. Perceived Stress

The response was measured through the 14-item Perceived Stress Scale (PSS) of Cohen. The mean PSS sum score for all respondents was 27.6 (SD = 8.3). Among respondents with non-missing PSS data, 54.9% were classified as stressed and 45.1% as not stressed. Missing PSS data accounted for approximately 16% of the sample. Respondents in their fourth year of studies reported lower perceived stress than those in the second and third years. Additionally, around 60% of the female respondents were more stressed than 50% of the male respondents. Sixty-two per cent of respondents who considered their financial resources insufficient were under perceived stress, the highest proportion in any given stress level category.

#### 4.2.3. Depressive Symptoms

The mean cumulative score on the Modified Beck Depression Inventory (M-BDI) was 33.8 (SD = 16.2). Among respondents with non-missing M-BDI data, 44.2% were classified as depressed and 55.8% as not depressed. Females were more depressed (48%) than their male counterparts (40%) (see Table 3). Finally, respondents who considered their financial resources insufficient were more depressed than those who did not.

### 4.3. Academic Performance

The data on academic performance showed that 23.9% of the respondents were in the lowest GPA category (≤3.00), and 25.2% were in the 3.01–3.30 category. A larger proportion of respondents (35.8%) fell in the 3.31–3.70 category, whereas 15.1% were in the highest GPA category (3.71–4.00) (see Table 3).

Only 16.6% of the female respondents had the lowest grades, compared with 30.9% of the male respondents. Regarding the original place of residence, around 30% of the rural respondents had the lowest grades, compared with around 20% of their urban counterparts.

In terms of subjective performance, 72.9% of respondents considered their academic performance better than their peers’, while 27.1% thought their performance was either the same or worse. There was variation in this measure across the faculties. A larger proportion of respondents from the Faculty of Social Sciences (81.4%) considered their academic performance better than those from the Faculty of Science (67.3%) and the Faculty of Commerce and Management (71.3%) (see Table 3). A large majority of students (91.4%) considered achieving good grades important, whereas only 7.5% did not. There were no substantial differences in responses across categories.

#### 4.3.1. Factors Associated with Perceived Mental Health Outcomes

Table 4 presents the factors associated with perceived stress, depressive symptoms, and low psychological well-being. Factors associated with the likelihood of experiencing mental health issues were somewhat different across different types of mental health issues. However, gender was one of those factors that influenced all types of mental health issues. Compared with female students, male students had lower odds of perceived stress OR = 0.74, 95% CI = 0.58–0.94), depressive symptoms (OR = 0.75, 95% CI = 0.58–0.96), and low psychological well-being (OR = 0.70, 95% CI = 0.53–0.93). Among BA/BSC students, those in the second year of their studies had a higher risk of PS (OR = 1.92, 95% CI = 1.18–3.12) than students in third and fourth years at the same degree level.

Students living in hostels were 50% less likely to experience LWB than those living at home. Students who had sufficient financial support were less likely to have any mental health issue than their counterparts, i.e., PS (OR = 0.70, 95% CI = 0.52–0.96), DS (OR = 0.53, 95% CI = 0.38–0.74), and LWB (OR = 0.53, 95% CI = 0.38–0.73).

The possible interpretation is the habit of denying negative feelings or thinking about mental issues as personal weaknesses, particularly in a case of academic or family environment where success and happiness are highly valued. This cultural setting can lead to an individual under-reporting their depressive symptoms or painting a rosier self-portrait than their mental condition warrants. Moreover, psychological well-being and depression came to be measured by the self-report surveys that did not cover the complexity of the concept of mental health. As an example, a person can tell that they enjoy life and feel some sense of accomplishment, yet still feel some lingering symptoms of depression, especially when there is a lot of pressure, like with an exam or academic pressure.

#### 4.3.2. Association Between Mental Health Issues and Academic Performance

Table 5 shows that academic performance categories differed significantly by sex and place of birth. Male students were more likely than female students to fall into the lower academic performance categories, while female students were more likely to be in the high grades and satisfied category. Similarly, rural students were more concentrated in the lower performance categories than urban students.

Table 6 shows that the clearest pattern was related to grade dissatisfaction, rather than low grades alone. Students with higher perceived stress and depressive symptoms had significantly greater odds of being in both the low grades and unsatisfied group and the high grades and unsatisfied group, compared with students who had high grades and were satisfied with their grades. In contrast, low psychological well-being was significantly associated only with the high grades and the unsatisfied group. The low grades and satisfied category were not significantly associated with perceived stress, depressive symptoms, or low psychological well-being. Overall, these findings suggest that mental health problems were more consistently linked with dissatisfaction with academic performance, especially among students who reported high grades but remained dissatisfied.

The findings from this study are aligned with the Stress-Coping Model, which suggests that stress arises when students perceive a mismatch between academic demands and their coping resources. Although coping strategies were not directly measured in this study, the observed patterns are broadly consistent with the Stress-Coping Model, which emphasizes the role of appraisal and coping processes in shaping mental health outcomes. Specifically, we found that students who reported lower academic satisfaction also reported higher levels of anxiety and depression. This supports the model’s concept of cognitive appraisal, where individuals assess whether they can cope with stress. In our case, students with greater academic dissatisfaction exhibited higher stress levels, suggesting that students’ mental health is deeply influenced by how they perceive their academic performance and manage the associated stress.

## 5. Discussion

In the context of growing concern about student mental health globally, this study provides evidence from Pakistan, showing that perceived stress and depressive symptoms are widespread among university students and are closely associated with negative academic experiences. The gender distribution in the sample was nearly equal and reflected the gender composition of students enrolled at the universities included in the study. Most respondents (61.5%) were from urban areas, which is consistent with broader enrollment patterns in Pakistan. This urban predominance may reflect lower educational access in rural areas due to limited educational facilities [26], patriarchal norms that affect female education [27,29], and lower access to mass media and information resources [30].

With regard to mental health, perceived stress and depressive symptoms were highly prevalent, whereas low psychological well-being was less common. In this study, the prevalence of perceived stress was 54%, which is higher than that reported in many studies conducted at Pakistani universities, where estimates have generally ranged from 25% to 58% [14,31,32]. Studies reporting lower prevalence were more likely to have used instruments other than the Cohen Perceived Stress Scale (PSS) applied in this study [33,34,35]. Another study from Pakistan reported a prevalence of 58% among medical students using the same PSS instrument [36]. International comparisons also show high levels of perceived stress among university students in Saudi Arabia [37], Ethiopia [38], Canada [39], and France [40], whereas lower levels have been reported in Germany, Poland, Bulgaria, and Denmark [40]. Consistent with findings from Egypt, Malaysia, India, the United States, and Europe [41], female students in the present study reported higher perceived stress than male students.

With respect to the academic year, second- and third-year students experienced greater stress than final-year students. This finding differs from a previous study that reported higher stress among final-year students [42]. Consistent with earlier research, students with insufficient financial resources reported higher levels of stress than their peers [43,44,45]. The prevalence of depressive symptoms in this study was 44%, which is at the upper end of the range reported in previous studies (15% to 46%) [46,47,48]. This finding is consistent with reports from Central and Eastern Europe [49] and the Middle East [50], where the prevalence of depressive symptoms was reported to be 43% and 45%, respectively. In contrast, lower prevalence has been reported in Western Europe, the United States, and Canada [38,51]. This pattern is broadly consistent with evidence suggesting that students in high-income countries may, overall, experience a lower burden of mental health problems [34,46].

In contrast to the high prevalence of perceived stress and depressive symptoms, low psychological well-being was observed in less than one-fifth of the students. This finding may reflect differences in measurement constructs and instruments. The WHO-5 Index, widely used to assess subjective well-being, may be less sensitive to certain psychological symptoms than other tools such as the PSS or M-BDI. These findings are in line with studies in the United States and Europe, which reported low well-being among a minority of university students [52].

Although most students reported high psychological well-being (81.0%), depressive symptoms were also common (44.2%). This apparent discrepancy suggests that well-being and depressive symptoms capture related but distinct dimensions of mental health. It may also reflect the influence of self-report bias and social desirability, particularly in contexts where mental health stigma is prevalent. Respondents may be more inclined to endorse positively framed well-being items (e.g., WHO-5), while underreporting symptoms captured through instruments such as the M-BDI. This pattern may also extend to subjective measures such as academic satisfaction and perceived performance, which are inherently more vulnerable to social desirability influences. Therefore, the observed associations between mental health indicators and academic dissatisfaction should be interpreted with caution, as both constructs may be affected by shared reporting bias rather than fully independent processes.

In the context of the Stress-Coping Model, cultural appraisals significantly influence coping mechanisms. Social desirability bias may affect how students perceive and report their mental well-being, particularly in a context where expressing distress is sometimes viewed as a sign of weakness. The model suggests that stressors such as academic pressure are interpreted through culturally specific lenses, which could shape the coping strategies students adopt in response to perceived mental health challenges.

Because the data are cross-sectional, the temporal ordering of mental-health problems and academic dissatisfaction cannot be established. Students experiencing stress or depressive symptoms may be more likely to feel dissatisfied with their academic performance; however, dissatisfaction with academic performance may also increase stress and depressive symptoms. These relationships may also be reciprocal or shaped by shared contextual factors such as financial strain, academic environment, and social support. Therefore, the findings should be interpreted as associations rather than causal effects.

A significant association between mental health issues and financial strain was observed in this study. Students with insufficient financial resources were more likely to report high stress and depressive symptoms. This finding aligns with studies conducted in other parts of the world, including those in India and Malaysia, where financial stress is linked to poor mental health outcomes in students [53,54]. In Pakistan, where many students rely on family support for their education, financial pressure can become a major source of distress. Similar results have been observed in European and North American studies, where financial difficulties are reported to exacerbate mental health issues among students, especially those from low-income backgrounds [55,56].

This study also found that mental health issues, particularly high stress and depressive symptoms, were more consistently linked with dissatisfaction with academic performance than with low grades alone. Students who experienced higher levels of perceived stress or depression were more likely to report dissatisfaction with their academic outcomes, regardless of their actual grades. This pattern mirrors findings from previous studies, where mental health problems such as anxiety and depression were found to significantly influence students’ academic satisfaction and performance [57,58]. Interestingly, while academic performance is often viewed in terms of grades, many students may still experience dissatisfaction even with high grades, indicating a deeper issue with emotional and psychological well-being [59].

These results further support the Stress-Coping Model, particularly the model’s differentiation between problem-focused coping and emotion-focused coping. Students who engaged in problem-focused coping, such as seeking academic help or improving time management, were better able to manage stress and maintain positive mental health. In contrast, those who leaned towards emotion-focused coping strategies, such as avoidance or denial, experienced poorer mental health outcomes. This distinction highlights the importance of addressing coping strategies as part of interventions aimed at improving mental well-being in students.

The data presented in this study were collected in 2015; it provides a critical historical perspective on the mental health issues faced by university students in Pakistan prior to the global pandemic. Given the substantial shifts in the global mental health landscape, particularly in the post-pandemic era, this study’s findings should be viewed as a baseline for understanding the pre-COVID state of mental health and academic satisfaction among students. Future research can use this data as a comparative reference to examine how the mental health and academic experiences of students have evolved in the aftermath of the pandemic [60].

Findings from this study are important; there are several limitations to consider. The cross-sectional design of this study means that causality cannot be inferred from the results. Longitudinal studies are needed to better understand how mental health problems and academic performance are related over time. Additionally, the study relied on self-reported data, which may be subject to response bias. Future studies could employ mixed-methods approaches to gather more nuanced insights into how students cope with mental health challenges and how these challenges impact their academic lives.

An important limitation of the current study is its cross-sectional design, which restricts our ability to draw conclusions about causal relationships. The associations identified in this study should be interpreted with caution, as reverse or reciprocal relationships may exist. Longitudinal studies are needed to explore the directionality of these relationships.

The dichotomization of mental-health scales facilitated prevalence estimation and logistic modeling but may have resulted in loss of information compared with continuous-score analysis.

Although established instruments were used to assess psychological well-being, perceived stress, and depressive symptoms, formal psychometric validation of the adapted measures in this specific population was limited. Given the lack of psychometric validation in the current context, the results should be interpreted with caution. Future studies should consider validating these measures in the target population to further ensure their reliability and cultural relevance.

The findings should be generalized primarily to students enrolled in public universities in Punjab, Pakistan. They may not necessarily represent students in private universities, students in other provinces, or out-of-university young adults.

Given the self-report nature of the mental health indicators, academic satisfaction, and academic performance measures, the findings may be subject to recall bias and social desirability bias, especially in contexts where mental health stigma exists. Future research could incorporate objective measures such as clinical interviews or behavioral data to reduce these biases [61,62].

This study primarily documents associations in a single regional context; it lays the foundation for future research exploring how academic satisfaction and performance impact mental health across different cultural and educational settings. Further comparative studies can use this research as a baseline for understanding how these dynamics shift in response to various interventions and evolving academic environments.

## 6. Conclusions

Our findings indicate a high prevalence of perceived stress and depressive symptoms among university students, whereas most respondents reported high psychological well-being. Mental health challenges were more consistently associated with dissatisfaction with academic performance than with low grades alone. These findings highlight the need for universities to strengthen institutional support for student mental health. Universities should expand accessible mental health services, including counseling and stress management programs, and implement awareness initiatives to reduce stigma surrounding mental health problems. Establishing comprehensive support systems that involve peer networks, faculty engagement, and professional mental health services may help students better manage psychological challenges and improve their academic experiences.

This study fills an important gap in the literature by investigating the relationship between mental health, academic satisfaction, and performance among university students in Punjab, Pakistan. By utilizing the Stress-Coping Model, we have shown how academic stress and coping mechanisms interact to influence students’ mental well-being. Our findings suggest that improving students’ academic satisfaction and coping strategies could play a crucial role in supporting their mental health. Future studies should focus on testing interventions that target coping mechanisms to improve both mental health outcomes and academic performance.

## Figures and Tables

**Table 1:** Sample size calculation.

No	Name of University	City	Enrollment	Proportion	Sample Size *n* = *N*/[1 + *N*(e)^2^]
**1**	University of the Punjab	Lahore	27,782	0.55	*n* = 50,549/1 + 50,549(0.03)^2^
**2**	Bahauddin-Zakria University	Multan	13,910	0.275	
**3**	University of Gujrat	Gujrat	8857	0.175	*n* = 1087
Total			50,549	1	1087

Source: Higher Education Commission, Pakistan [28].

**Table 2:** Socio-demographic characteristics of students in the survey in Punjab, Pakistan (*N* = 1308).

Variable	Frequency	Percentage
Age (in four groups)		
≤20	366	28.0%
21	319	24.4%
22	321	24.5%
≥23	302	23.1%
Sex		
Male	657	50.2%
Female	651	49.8%
Place of birth		
Rural	466	35.6%
Urban	805	61.5%
Marital status		
Un-married	1247	95.3%
Married	45	3.4%
Divorced	2	0.2%
Separated	6	0.5%
BMI-group data		
Underweight	268	20.5%
Normal weight	728	55.7%
Overweight	167	12.8%
Obesity	57	4.4%
Current employment status		
Unemployed	1166	89.1%
Employed	113	8.6%
Religion		
Muslim	1294	98.9%
Non-Muslim	8	0.6%

**Table 3:** Frequencies and percentages of well-being, perceived stress, depression, academic grades, and academic performance.

		Frequency	Percentage
Well-being	Low	243	19.0%
	High	1034	81.0%
	Total	1277	100.0%
Perceived Stress	Not stressed	499	45.1%
	Stressed	607	54.9%
	Total	1106	100.0%
Depression	Not depressed	551	55.8%
	Depressed	436	44.2%
	Total	987	100.0%
Academic grades (GPA)	≤3.00	302	23.9%
	3.01–3.30	319	25.2%
	3.31–3.70	453	35.8%
	3.71–4.00	191	15.1%
	Total	1265	100.0%
Performance at the university in comparison to fellow students	Better	934	72.9%
	Same	194	15.1%
	Worse	153	11.9%
	Total	1281	100.0%

**Table 4:** Factors associated with perceived prevalence of mental health vs. non-prevalence of mental health issues (*N* = 1308).

Respondent Characteristics	Mental Health Issues		
	Perceived Stress	Depressive Symptoms	Psychological Well-Being
	OR (95% CI)	OR (95% CI)	OR (95% CI)
Age (Yrs)			
≤20	1.39 (0.99–1.94)	1.22 (0.85–1.73)	1.17 (0.79–1.74)
21	1.09 (0.78–1.54)	1.08 (0.75–1.55)	1.60 (0.77–1.74)
22	1.08 (0.77–1.52)	0.74 (0.51–1.07)	0.89 (0.58–1.35)
≥23	1.00	1.00	1.00
Sex			
Male	0.74 (0.58–0.94) **	0.75 (0.58–0.96) *	0.70 (0.53–0.93) *
Female	1.00	1.00	1.00
Marital Status			
Un-married	1.37 (0.73–2.56)	0.51 (0.27–0.97) *	0.60 (0.32–1.14)
Ever-married	1.00	1.00	1.00
Place of birth			
Rural	0.90 (0.70–1.16)	0.87 (0.66–1.13)	0.91 (0.67–1.23)
Urban	1.00	1.00	1.00
Monthly family income			
≤20,001	1.15 (0.73–1.79)	1.02 (0.62–1.59)	0.90 (0.54–1.49)
20,000–40,000	1.03 (0.67–1.48)	0.96 (0.63–1.46)	0.83 (0.53–1.31)
40,001–60,000	1.13 (0.75–1.70)	0.94 (0.61–1.44)	0.76 (0.48–1.22)
60,001–80,000	0.874 (0.51–1.74)	0.998 (0.58–1.70)	0.69 (0.37–1.30)
≥80,000	1.00	1.00	1.00
Employment status			
Unemployed	1.23 (0.82–1.86)	0.96 (0.62–1.48)	1.11 (0.66–1.86)
Employed	1.00	1.00	1.00
University			
Bahauddin Zakariya University	0.92 (0.69–1.22)	0.66 (0.48–0.91) **	0.68 (0.47–0.97) *
University of Gujrat	1.05 (0.74–1.35)	0.94 (0.69–1.28)	1.01 (0.71–1.43)
University of the Punjab	1.00	1.00	1.00
Academic Faculty			
Social Sciences	1.06 (0.80–1.40)	1.23 (0.91–1.65)	1.33 (0.96–1.84)
Commerce and Management	1.01 (0.75–1.35)	1.06 (0.77–1.45)	0.98 (0.68–1.40)
Science	1.00	1.00	1.00
Degree/programme			
B.A/B.S (Hons)	1.15 (0.90–1.46)	1.33 (1.03–1.71) *	1.22 (0.92–1.61)
Master	1.00	1.00	1.00
Type of your degree			
Regular (morning)	0.79 (0.62–1.01)	0.90 (0.70–1.17)	1.25 (0.94–1.68)
Self-support (evening)	1.00	1.00	1.00
Year of study			
2nd year	1.92 (1.18–3.12) **	1.02 (0.61–1.69)	1.55 (0.80–2.98)
3rd year	1.74 (1.01–3.03) *	1.13 (0.63–2.02)	2.03 (0.99–4.15)
4th year	1.00	1.00	1.00
Current place of living			
University hostel	0.82 (0.61–1.10)	0.78 (0.57–1.07)	0.50 (0.34–0.74) **
Private hostel	0.63 (0.43–0.92) *	0.69 (0.46–1.04)	0.57 (0.25–0.94) *
Home	1.00	1.00	1.00
Sufficiency of Financial Support			
Fully sufficient	0.70 (0.52–0.96) *	0.53 (0.38–0.74) ***	0.53 (0.38–0.73) ***
Insufficient	1.00	1.00	1.00

Notes: OR = Odds ratio; CI = Confidence interval. * *p* < 0.05; ** *p* < 0.01; *** *p* < 0.001. A simple binary logistic regression analysis was employed separately with each type of mental health issue.

**Table 5:** Association between respondent characteristics and mental issues with academic performance.

Respondent Characteristics	Academic Performance				
	Low Grades and Unsatisfied with Grades	Low Grades and Satisfied with Grades	High Grades and Unsatisfied with Grades	High Grades and Satisfied with Grades	*p*-Value *
Sex					
Male	57.4%	57.0%	48.6%	42.0%	0.000
Female	42.6%	43.0%	51.4%	58.0%	
Total	100.0%	100.0%	100.0%	100.0%	
Place of birth					
Rural	41.9%	38.3%	31.2%	32.7%	0.026
Urban	58.1%	61.7%	68.8%	67.3%	
Total	100.0%	100.0%	100.0%	100.0%	

* *p*-value is based on the chi-square statistic.

**Table 6:** Association between Mental Health Issues and Academic Performance (Multinomial Logistic Regression Model).

Mental Health Issues	Academic Performance		
	Low Grades and Unsatisfied with Grades	Low Grades and Satisfied with Grades	High Grades and Unsatisfied with Grades
	AOR (95% CI)	AOR (95% CI)	AOR (95% CI)
Perceived Stress			
Stressed	1.66 (1.19–2.31) **	1.15 (0.82–1.62)	1.72 (1.16–2.55) **
Not Stressed	1.00	1.00	1.00
Depressive symptoms			
Depressed	1.56 (1.10–2.20) *	0.81 (0.56–1.19)	1.98 (1.30–3.02) **
Not depressed	1.00	1.00	1.00
Psychological well-being			
Low	1.29 (0.88–1.90)	0.74 (0.47–1.15)	1.67 (1.09–2.55) *
High	1.00	1.00	1.00

Notes: AOR = adjusted odds ratio; CI = 95% confidence interval. Adjusted odds ratios were estimated controlling for sex, place of birth, university, academic faculty, degree/program, and current place of living. * *p* ≤ 0.05; ** *p* ≤ 0.01.

## Data Availability

The data supporting the reported results in this study can be obtained from the corresponding author upon reasonable request.

## Abbreviations

The following abbreviations are used in this manuscript:

CNSHS	Cross-National Student Health Study
DALYs	Disability Adjusted Life Years
DS	Depressive Symptoms
HEC	Higher Education Commission
HIV/AIDS	Human Immunodeficiency Virus/Acquired Immunodeficiency Syndrome
M-BDI	Modified Beck Depression Inventory
PSS	Perceived Stress Scale
PWBI	Psychological Well-Being Index
WHO	World Health Organization
YLDs	Years Lived with Disability
